# Basic psychological needs satisfaction, intrinsic motivation, and studying sports protect university student-athletes from burnout: insights from Latvia

**DOI:** 10.3389/fspor.2025.1548583

**Published:** 2025-08-12

**Authors:** Svens Vilks, Aleksandrs Kolesovs

**Affiliations:** ^1^ Department of Anatomy, Physiology, Biochemistry, Biomechanics, Hygiene and Informatics, Riga Stradins University, Riga, Latvia; ^2^ Department of Psychology, University of Latvia, Riga, Latvia

**Keywords:** basic psychological needs, intrinsic motivation, burnout, university student-athletes, competition level

## Abstract

The study explored the prediction of athlete burnout by basic psychological needs satisfaction in sports, combined with the intrinsic motivation in sports and sociodemographic characteristics of university student-athletes. The protective effects of both psychological predictors were expected. The participants were 219 university student-athletes aged 19–33 (M = 22.3 and SD = 3.2 years) who participated in sports from recreational (38%) up to World/Olympic (9%) levels, with a mean sports experience of 10.1 (SD = 5.2) years. The study used three surveys, presented in Latvian: Sports Motivation Scale-II, Basic Psychological Needs in Exercise Scale, and Athlete Burnout Questionnaire. Higher need satisfaction was the main predictor of lower burnout and a higher sense of accomplishment in the frame of SEM. Higher intrinsic motivation, participants' age, and studying sports also predicted lower burnout and competing at World/Olympic level predicted a higher sense of accomplishment. The bifactor model confirmed athlete burnout as a syndrome integrating its specific components. As the main protective factor, psychological needs satisfaction in sports should be prioritized in university student-athlete training.

## 1 Introduction

Participation in sports provides athletes with essential physical and mental health benefits, contributing to long-term well-being and resilience. However, athlete burnout—a psychological syndrome defined by emotional and physical exhaustion, reduced sense of accomplishment, and devaluation of one's sport (1)—remains a serious issue in the athletic community, particularly among student-athletes who juggle dual responsibilities. Burnout often leads to disengagement from sports and can have a prolonged negative impact on both athletic and non-athletic domains of life (2).

A meta-analytical study (3) indicates a worrying increase in burnout symptoms among athletes from 1997 to 2019, with significant rises in feelings of reduced accomplishment and sport devaluation. Factors contributing to this rise include heightened expectations from coaches and parents, scholarship pressures, and lack of autonomy within training environments (4, 5). Such demands can intensify stress, particularly when combined with unsupportive environments that fail to meet athletes' basic psychological needs [e.g., (6)]. Studies stress that while some athletes thrive under pressure, others may face burnout due to an interplay of individual resilience and environmental demands (7). Anxiety and mental health indicators, such as perceived stress, have also been identified as mediating factors, highlighting the complexity of burnout risk in competitive settings (8, 9).

Furthermore, the COVID-19 pandemic has introduced additional complexity to the issue of athlete burnout (10, 11). The pandemic disrupted training and competition schedules, increased uncertainty, and, in some cases, reduced access to social and psychological support. Recent studies suggest that while some athletes experienced heightened stress and risk of burnout due to these disruptions, others reported reduced burnout symptoms due to decreased competitive demands and increased recovery time (10, 12). Therefore, the impact of the pandemic on athlete burnout may not align directly with pre-pandemic trends.

The Self-Determination Theory (SDT) provides a robust framework for understanding burnout risk factors and preventive mechanisms, emphasizing the importance of intrinsic motivation and the satisfaction of basic psychological needs—autonomy, competence, and relatedness (13). Satisfying these needs is critical in preventing burnout, as supportive environments help to maintain athletes' engagement and psychological health. Meta-analyses demonstrate that basic need satisfaction and intrinsic motivation are among the protective factors for athletes' burnout (14), and SDT-based interventions can positively impact health behaviors and psychological outcomes by fostering motivation, especially its intrinsic and autonomous forms (15, 16).

Furthermore, the importance of autonomy support is highlighted in reducing burnout. When coaches provide autonomy-supportive guidance, they foster environments that protect against psychological distress by satisfying athletes' needs for independence, competence, and social connectedness (17). Studies also show that higher satisfaction of competence and relatedness is associated with lower burnout rates, while frustration of these needs exacerbates burnout risk (18, 19).

Intrinsic motivation, a key aspect of motivational regulation, acts as a powerful protective factor against burnout (14). Athletes driven by personal satisfaction rather than external rewards tend to be more engaged in sports (20), whereas reliance on extrinsic motivation, while effective in enhancing performance, can increase pressure and anxiety, potentially contributing to burnout if not balanced with intrinsic motivation (21). Moreover, competitive level influences burnout risk, with elite athletes facing increased pressures and higher burnout rates (22, 23).

Given the complex interplay between individual and environmental factors, the development of supportive, need-fulfilling environments in sports settings remains essential for mitigating burnout. Accounting for recent findings [e.g., (24)], we considered intrinsic motivation as a mediator in the relationship between athletes' basic needs satisfaction and burnout. In addition, we endorsed a comparison of bifactor models of psychological constructs under consideration with other factorial models (univariate, multi-component, and a higher-order factor) to add to the discussion on general models of burnout [e.g., (25)] and needs satisfaction in sports [e.g., (26)]. Therefore, this study aimed at a complex assessment of athletes' needs satisfaction, intrinsic motivation in sports, and environmental factors (including the level of competition, demographics, and studying sports) as predictors of their burnout within a model involving intrinsic motivation as a mediator of the effect of need satisfaction.

## 2 Material & methods

### 2.1 Participants and procedures

The Ethics Committee of the Medical Faculty of the University of Latvia (Nr. 19-25/114) approved the study, which ensured that all procedures correspond with current legal and data safety requirements. Moreover, the study was carried out in line with the Declaration of Helsinki and its later amendments.

Participants were recruited from higher education institutions in Latvia. The primary investigator sought contact with institutions through the institution's sports clubs and their respected sports teams and coaches. Students were then approached by sending a personal email from the primary investigator. All students obtained information about the general goals of this study and were informed that participation was voluntary, and that data would be treated confidentially. Students were also informed that not participating in the study had no consequences. Before the beginning of the data collection, participants were asked to read the instructions carefully. In accordance with them, completing the questionnaire confirmed the participant's consent to data analysis. The instructions again stated that participation is voluntary, and participants do not have to submit their answers if they do not want to. The data collection period lasted from April to May 2022. All participants filled in a questionnaire with a battery of internationally accepted psychological instruments.

The minimum sample size was calculated online (27) for nine latent variables, 37 observed indicators, an alpha level of 0.05, a statistical power of 0.95, and an anticipated effect size of 0.43 (14). Calculations resulted in a minimum sample size of 103, which was corrected to at least 200 participants. Table 1 represents the demographic characteristics of 219 students aged 19–33 who participated in sports recreationally or at various competition levels.

**Table 1:** Demographic characteristics of participants (*N* = 219).

Demographic variable	Number of participants (%)	M (SD)
1. Gender		
1.1. Female	136 (62%)	
1.2. Male	83 (38%)	
2. Age, years		22.3 (3.2)
2. Domain of studies		
2.1. Sports-related (Sports Pedagogy or Sports Science)	124 (57%)	
2.2. Other	95 (43%)	
3. Experience in sports, years		10.1 (5.2)
4. Sports type		
4.1. Individual	115 (52%)	
4.2. Team	104 (48%)	
5. Competition level		
5.1. Recreational	83 (38%)	
5.2. National	69 (32%)	
5.3. International	47 (21%)	
5.4. World Championships/Olympics	20 (9%)	

### 2.2 Measures

Basic psychological needs were assessed with a translated version of the Basic Psychological Needs in Exercise Scale (BPNES) (28), translated into the Latvian language by the authors following a parallel translation procedure. The scale is theoretically grounded in the self-determination theory (SDT) and consists of 11 items grouped into three subscales of basic psychological needs: autonomy, including four items (e.g., “The exercise program I follow at the facility is aligned with my interests.”), competence, including four items (e.g., “I have made great progress as far as the result pursued is concerned.”), and relatedness, including three items (e.g., “I feel very comfortable when I do exercise with other participants.”). This questionnaire used a Likert-type scale ranging from (1) “totally disagree” to (5) “totally agree”.

Intrinsic motivation was assessed with the Sports Motivation Scale-II (SMS-II) (29), also translated by the authors following a parallel translation procedure. The SMS-II is also theoretically grounded in the SDT and assesses individuals' levels of motivation toward sport and exercise. It consists of 18 items equally distributed into six factors/motivation types (intrinsic regulation, integrated regulation, identified regulation, introjected regulation, external regulation, and amotivated regulation). Respondents answered on a 7-point Likert scale ranging from (1) “does not correspond at all” to (7) “corresponds completely”.

Athlete burnout was assessed with the translated Latvian version (30) of the Athlete Burnout Questionnaire (ABQ) (1). The ABQ is a self-report inventory that consists of 15 items. Respondents used a 5-point Likert scale, with the following stem “How often do you feel this way?” and the anchors: (1) almost never, (2) rarely, (3) sometimes, (4) frequently, and (5) almost always. Three scores were obtained by calculating means over the items equally distributed into three subscales: emotional/physical exhaustion (e.g., “I feel overly tired from my sport participation.”), sport devaluation (e.g., “I don’t care as much about my sport performance as I used to do.”), and reduced sense of accomplishment (e.g., “I am not performing up to my ability in sport.”). Items 1 and 14 were inverted before calculating the subscale mean scores.

### 2.3 Statistical analysis

IBM SPSS Statistics for Windows 22.0 was applied for regular statistical tests. The “lavaan” 0.6–11 (31) package for R Project for Statistical Computing was used for the confirmatory factor analysis and structural equation modeling (SEM). The robust diagonally weighted least squares (DWLS) method was applied to estimate data from Likert-type scales. The “BifactorIndicesCalculator” 0.2.2 package (32) was applied to calculate the bifactor model's indices (33).

## 3 Results

The correlation analysis indicated significant relationships among the variables under consideration (Table 2). Correlations among components of athlete burnout and need satisfaction indicated their positive relationships and possible general factors for both psychological constructs. The significant negative correlations of athlete burnout with need satisfaction and intrinsic motivation informed the following use of SEM to assess complex interrelations among variables.

**Table 2:** Descriptive statistics, reliability, and zero-order correlations among athlete burnout, satisfaction of needs, and intrinsic motivation (*N* = 219).

Scale	M (SD)	K	*α*	1.	1.1.	1.2.	1.3.	2.	2.1.	2.2.	2.3.
1. Burnout sum	36.35 (7.91)	15	.81	–							
1.1. Exhaustion	11.45 (3.63)	5	.82	.79***	–						
1.2. Accomplishment (R)	13.47 (3.52)	5	.73	.69***	.28***	–					
1.3. Devaluation	11.44 (3.34)	5	.63	.78***	.49***	.29***	–				
2. Need satisfaction sum	41.64 (6.37)	11	.83	−.44***	−.23**	−.52***	−.25***	–			
2.1. Autonomy	14.93 (2.95)	4	.69	−.39***	−.23**	−.40***	−.24***	.87***	–		
2.2. Competence	15.19 (2.64)	4	.71	−.41***	−.16*	−.54***	−.23**	.82***	.61***	–	
2.3. Relatedness	11.53 (2.36)	3	.80	−.25***	−.14*	−.30***	−.13	.70***	.41***	.33***	–
3. Intrinsic motivation	18.35 (3.26)	3	.82	−.21**	−.20**	−.07	−.20**	.15*	.17*	.15*	.01

R, reduced; K, number of items; *Α*, Cronbach's alpha.

* *p* < .001.

** *p* < .01.

*** *p* < .05.

An assessment of factor structure of athlete burnout and need satisfaction in the exercise included four models: (1) the unidimensional model, considering all items loaded by a single factor; (2) the correlated factor model, presenting the construct as multidimensional; (3) the higher-order factor, mediated by first-level components; (4) the bifactor model, including the general factor and specific factors, associated with components of each construct. The confirmatory factor analysis (Table 3) demonstrated that the bifactor model fits the data best for both psychological constructs. For need satisfaction, correlated factors and a higher-order factor also demonstrated a good fit. Item 5 was the leading item in a specific autonomy factor, which demonstrated a negative variance estimate. Its covariance was constrained to zero, and the bifactor model was selected to test the general component of need satisfaction in the prediction of burnout.

**Table 3:** Fit indices of factorial models of the athlete burnout and need satisfaction in exercise (*N* = 219).

Model	χ^2^	df	*p*	CFI	TLI	RMSEA (90% CI)	PRMSEA <0.05	SRMR
Athlete burnout								
1	297.56	90	.000	0.85	0.83	0.103 (0.090, 0.116)	.000	0.108
2	184.34	87	.000	0.93	0.92	0.072 (0.057, 0.086)	.008	0.084
3	184.34	87	.000	0.93	0.92	0.072 (0.057,0.086)	.008	0.084
4	58.64	75	.918	1.00	1.00	0.000 (0.000, 0.015)	.999	0.047
Need satisfaction in exercise								
1	122.85	44	.000	0.93	0.91	0.091 (0.072, 0.110)	.000	0.095
2	40.95	41	.473	1.00	1.00	0.000 (0.000, 0.046)	.970	0.052
3	40.95	41	.473	1.00	1.00	0.000 (0.000, 0.046)	.970	0.052
4	31.94	34	.569	1.00	1.00	0.000 (0.000, 0.045)	.973	0.046

1 – Unidimensional. 2 – Correlated factors. 3 – Higher-order factor. 4 – Bifactor.

An additional analysis assessed the usefulness of specific factors within the model under consideration. The general factor of need satisfaction was described by ECV = 0.73, PUC = 0.67, and ω_H_ = 0.74 that confirmed its significance (33). This factor was supplemented by a specific factor of relatedness (ω_HS_ = 0.60), while other specific factors demonstrated hierarchical reliability lower than 0.20. The general factor of burnout demonstrated ECV = 0.47, PUC = 0.71, and ω_HS_ = 0.65, indicating significance of specific components within the model. Therefore, the sense of reduced accomplishment was added to the general factor because of a relatively high hierarchical reliability (ω_HS_ = 0.59). Other specific factors demonstrated hierarchical reliability under 0.30.

**Figure 1:**
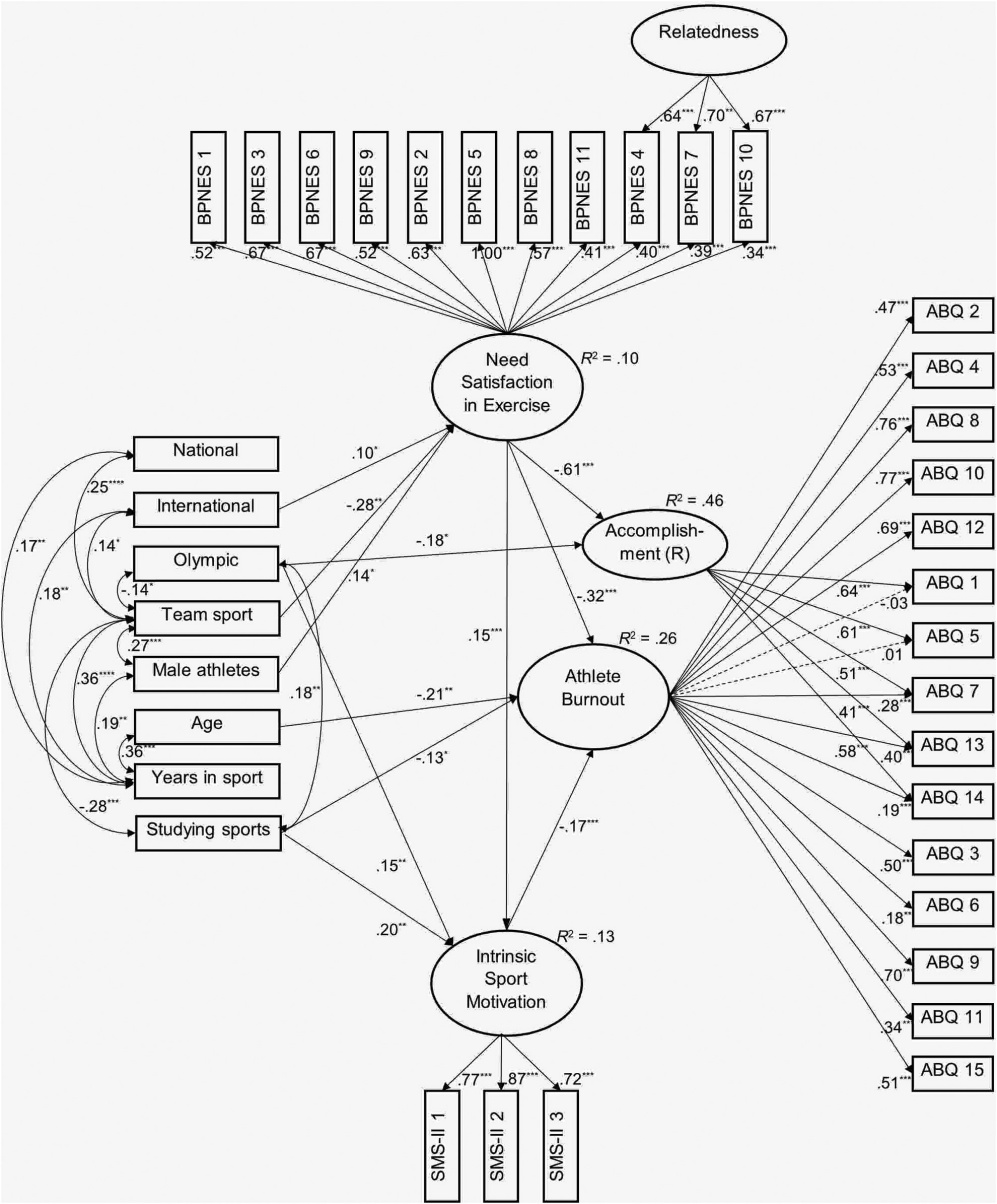
Predicting athlete burnout by need satisfaction in exercise, intrinsic motivation, level of competition, and demographics (*N* = 219. ABQ, The Athlete Burnout Questionnaire; BPNES, The Basic Psychological Needs in Exercise Scale; R, Reduced; SMS-II, Sport Motivation Scale II. Only significant standardized regression coefficients and covariances are presented. There were no covariances among dummy variables for the level of competition. Dashed lines present non-significant factor loadings. *** *p* < .001, ** *p* < .01, * *p* < .05).

The SEM of predicting burnout and the sense of reduced accomplishment by need satisfaction, relatedness, intrinsic motivation, and demographic variables demonstrated an acceptable model fit (34): χ^2^(558) = 703.54, *p* < .001, CFI = 0.963, TLI = 0.956, RMSEA = 0.035, 90%CI (0.026; 0.042), p_RMSEA_ _<_ _0.05_ = 1.000, and SRMR = 0.067. This model explained 26% of the variance of athlete burnout and 46% of the sense of reduced accomplishment (Figure 1).

The analysis revealed that the general factor of need satisfaction was the strongest predictor of lower athlete burnout (*β* = −0.32) and the sense of reduced accomplishment (*β* = −0.61) and positively predicted intrinsic motivation (*β* = 0.15). Both, the direct (*β* = −0.35, *z* = −8.69, *p* < 0.001) and indirect (*β* = −0.03, *z* = −3.11, *p* = 0.002) protective effects of athletes' basic needs satisfaction were significant in predicting burnout through intrinsic motivation is sports. Athletes’ age (*β* = −0.21), studying sports (*β* = −0.13), and intrinsic motivation (*β* = −0.17) also were predictors of lower burnout. Participation at the Olympic level protected from the sense of reduced accomplishment (*β* = −0.18) and positively predicted intrinsic motivation (*β* = 0.15). Studying sports also predicted intrinsic motivation (*β* = 0.19). The international level of competition (*β* = 0.10) and male gender (*β* = 0.14) positively predicted satisfaction of needs in exercise. In contrast, participation in team sports (*β* = −0.28) predicted athletes’ needs satisfaction negatively. The specific factor of relatedness remained without significant links to other variables.

Significant correlations among predictors indicated positive relationships between Olympic level and studying sports (*r* = 0.18). Additionally, two demographic variables were significantly related to other demographics. First, participation in team sports was positively associated with the national level of competition (*r* = 0.25), international level of competition (*r* = 0.14), years in the sport (*r* = 0.36), and male gender (*r* = 0.27). It is also negatively associated with participation at the Olympic level (*r* = −0.14) and studying sports (*r* = −0.28). Second, years in the sport are positively associated with age (*r* = 0.36), participation at the national (*r* = 0.17) and international (*r* = 0.18) levels of competition, and male gender (*r* = 0.19).

## 4 Discussion

The established model confirms complex links among observed variables: basic psychological needs satisfaction, intrinsic motivation, athletes' age, and studying sports demonstrate direct protective effects on athletes' burnout. Moreover, the best-fitting bifactor model supported the view of burnout as a syndrome and an additional value of reduced accomplishment as a specific factor [e.g., (25)]. Basic psychological needs satisfaction was the main predictor of lower burnout and a protective factor against a reduced sense of accomplishment. These findings emphasize the central role of needs satisfaction in mitigating burnout, which concurs with previous research (14, 18, 19) and theoretical assumptions of self-determination theory (13). The positive protective effect of intrinsic motivation also aligns with previous findings (15, 16). In addition, there was a very small indirect effect of athletes' needs satisfaction, which is conceptually in line with Wu et al. (24). A relatively low strength of the relationship between intrinsic motivation and needs satisfaction confirms their independent contribution to lowering burnout. Simultaneously, a comparison of predictive links suggests that focusing on needs satisfaction might have a more pronounced impact on solving the increasing problem of burnout in sports (3).

Sociodemographic factors also had effects within the predictive model. The observed protective effect of age can be twofold. On the one hand, athletes' experience dealing with burnout-associated challenges increases with age. On the other hand, more resilient athletes can demonstrate a higher commitment to sports. From a practical perspective, it might indicate a need for higher support for younger athletes in dealing with sports challenges. In addition, our findings confirm that a higher level of support is required for university student-athletes in fields unrelated to sports, as demonstrated previously at the school level [e.g., (7)].

Among sport-specific characteristics, a positive effect of the international level of competition on need satisfaction combined with an absence of a similar effect for athletes participating in world championships and Olympic games. In turn, the highest level of competition, protected from a sense of reduced accomplishment and associated with intrinsic motivation, supporting engagement in sports [e.g., (20)]. Observed relationships between the level of competition and protective factors indicate a nonlinear relationship between the level of competition and need satisfaction with a changing trend among elite athletes. Therefore, the step to international competitions seems a threshold for satisfaction in sports in our sample, and participation at the elite level is associated with the highest intrinsic motivation and the sense of accomplishment.

Another effect of sport-specific characteristics was observed in athletes from team sports. They demonstrated a lower level of basic need satisfaction in sports, which indicates a need for supporting athletes' autonomy and competence combined with fostering their relatedness within a team, as confirmed by the analysis of previous studies (6, 14).

### 4.1 Limitations and future directions

A relatively small number of participants forms a substantial limitation of the study. Therefore, the observed effects should be further investigated. Simultaneously, for a relatively small country such as Latvia, it involved student-athletes at a maximal range of the level of competition, including the Olympics. Another limitation relates to the statistical perspective on the relationships under consideration. We selected the best statistical solution (a bifactor model) for the models of basic need satisfaction and burnout. A more detailed comparison of this model with multi-component models, including a broader spectrum of motivation, is in question under larger sample sizes due to the need for invariance testing (26). In addition to these limitations, the cross-sectional design limits any assessment of the dynamics of indicators under consideration. Therefore, a cross-lagged study will help answer the question regarding the interaction between variables in time, considering possible changes after the COVID-19 pandemic.

### 4.2 Theoretical contribution and practical applications

It should be noted that the study contributes to the literature on athlete burnout. From a theoretical perspective, we contributed to debates about general and specific components of athletes' need satisfaction and burnout [e.g., (25)]. In addition, we provided additional evidence for the partial mediating role of intrinsic motivation in the relationship between needs satisfaction and burnout (24) in university students. Practically, we identified sociodemographic and sport-specific predictors of burnout, which could inform targeted interventions to support athletes' well-being, particularly in non-sport educational fields and team sports.

### 4.3 Conclusions

In summary, the results emphasize the protective role of basic psychological needs satisfaction in dealing with athlete burnout with intrinsic motivation, international competition level, and age, providing additional protective effects. Prioritizing athletes' psychological needs and supporting intrinsic motivation could be highly significant for younger athletes, team sports participants, and university student-athletes in non-sport educational fields.

## Data Availability

The original contributions presented in the study are included in the article/Supplementary Material, further inquiries can be directed to the corresponding author.
